# Liquid-Liquid Phase Separation Phenomenon on Protein Sorting Within Chloroplasts

**DOI:** 10.3389/fphys.2021.801212

**Published:** 2021-12-24

**Authors:** Canhui Zheng, Xiumei Xu, Lixin Zhang, Dandan Lu

**Affiliations:** State Key Laboratory of Crop Stress Adaptation and Improvement, School of Life Sciences, Henan University, Kaifeng, China

**Keywords:** chloroplast, protein sorting, STTs, liquid-liquid phase separation, liquid droplets

## Abstract

In higher plants, chloroplasts are vital organelles possessing highly complex compartmentalization. As most chloroplast-located proteins are encoded in the nucleus and synthesized in the cytosol, the correct sorting of these proteins to appropriate compartments is critical for the proper functions of chloroplasts as well as plant survival. Nuclear-encoded chloroplast proteins are imported into stroma and further sorted to distinct compartments via different pathways. The proteins predicted to be sorted to the thylakoid lumen by the chloroplast twin arginine transport (cpTAT) pathway are shown to be facilitated by STT1/2 driven liquid-liquid phase separation (LLPS). Liquid-liquid phase separation is a novel mechanism to facilitate the formation of membrane-less sub-cellular compartments and accelerate biochemical reactions temporally and spatially. In this review, we introduce the sorting mechanisms within chloroplasts, and briefly summarize the properties and significance of LLPS, with an emphasis on the novel function of LLPS in the sorting of cpTAT substrate proteins. We conclude with perspectives for the future research on chloroplast protein sorting and targeting mechanisms.

## Introduction

Chloroplasts are essential semi-autonomous organelles that are primarily responsible for photosynthesis as well as many other functions, such as synthesis of amino acids, fatty acids, pigments, and hormones (Jarvis and Lopez-Juez, 2013). Chloroplasts contain highly complex sub-organellar compartments, including three membrane systems (the outer and inner envelope membrane and the thylakoid membrane) delineating three aqueous compartments (the intermembrane space, the stroma, and the thylakoid lumen) (Kirchhoff, 2019). Chloroplasts are estimated to contain approximately 3000 different proteins, but only about 100 proteins are encoded by the chloroplast genome (Jarvis and Robinson, 2004; Bouchnak et al., 2019). The vast majority of proteins are encoded in the nucleus and synthesized in the cytosol as precursors before being imported into chloroplasts post-translationally. Upon arrival at the stroma, some proteins reside and function therein, while other proteins are further sorted and targeted to the inner envelope or thylakoids depending on their specific targeting signals. Previous investigations have revealed different sorting pathways and numerous factors involved in intra-chloroplast protein sorting and targeting (Jarvis and Lopez-Juez, 2013; New et al., 2018; Ziehe et al., 2018; Xu et al., 2021a). However, how the targeting signals are recognized and how the substrates are sorted in the stroma are not yet fully elucidated.

Liquid-liquid phase separation (LLPS) is a basic physicochemical phenomenon, referred to a state transition in which a homogeneous liquid spontaneously de-mixes into two or more coexisting liquids (Alberti, 2017). However, recent investigations have shown that LLPS is a universal organizing principle of liquid condensates or membrane-less compartments in living cells, offering an exciting novel mechanism for intracellular organization (Brangwynne et al., 2009; Shin and Brangwynne, 2017). Intriguingly, LLPS has also been found within the chloroplast to facilitate formation of intraorganellar liquid droplets for protein sorting to thylakoid lumen (Ouyang et al., 2020).

In this review, we provide an overview of the current knowledge of protein sorting within chloroplasts, and briefly summarize the significance and key components of liquid condensates formed by LLPS, with a focus on the function of LLPS on protein sorting within the chloroplast. Finally, we prospect for the future research on intra-chloroplast protein sorting and targeting.

## Protein Sorting Within the Chloroplast

Nuclear-encoded chloroplast proteins are synthesized in the cytoplasm as precursors with an N-terminal cleavable targeting signal, known as the transit peptide (Bruce, 2000; Lee and Hwang, 2021). The transit peptide is recognized by the chloroplast protein import machinery, translocons at the outer (TOC) and inner (TIC) envelope membrane of chloroplasts, which facilitate the translocation of the protein across the outer and inner envelope. Some chloroplast inner membrane proteins, such as albino or pale green mutant 1 (APG1), are laterally released and thus inserted into the membrane by stop-transfer mechanism during translocation through TIC translocon (Viana et al., 2010; Lee et al., 2017). For other proteins, the N-terminal transit peptide is removed by the stroma processing peptidase (SPP) (Richardson et al., 2014). These proteins may stay in the stroma or be further sorted to other sub-organellar compartments, including the inner envelope membrane, thylakoid membrane, or thylakoid lumen (Jarvis and Lopez-Juez, 2013; Paila et al., 2015). Some inner envelope proteins, such as FTSH12 and TIC40, are translocated by the translocase SEC2 (Li et al., 2015, 2017; Lee et al., 2017), whereas thylakoid proteins are sorted and targeted by distinct pathways depending on the associated chaperones and energy required.

Thylakoid membrane-located proteins may be sorted by the following pathways: the chloroplast signal recognition particle (RP) pathway, the chloroplast Guided Entry of Tail-anchored protein (cpGET) pathway, or the “spontaneous insertion” pathway. The cpSRP pathway is involved in the insertion of light-harvesting chlorophyll-binding proteins (LHCPs) into thylakoid membranes. The 14 amino-acid sequence located at the beginning of the third transmembrane domain (TMD3) of LHCPs, T14, is recognized by a stromal ankyrin protein LHCP TRANSLOCATION DEFECT (LTD), which interacts with cpSRP43 and subsequently routes LHCPs from the TIC translocon to the cpSRP pathway (Ouyang et al., 2011). The unique plastid chaperone cpSRP43 binds LHCPs with their unique motif between TMD2 and TMD3, named the L18 peptide, and together with the cpSRP54 GTPase, protects the substrates from aggregation in the aqueous stroma (Stengel et al., 2008; Falk and Sinning, 2010). Subsequently, cpSRP43 and cpSRP54 interact with the membrane receptor CHLOROPLAST FILAMENTOUS TEMPERATURE SENSITIVE Y (cpFtsY), and the latter mediates the membrane insertion of LHCPs by integrase Albino3 (Abl3) in a GTP-dependent manner (Lee et al., 2017; Ziehe et al., 2018).

Chloroplast tail-anchored (TA) proteins, which possess a stroma-exposed N-terminus proceeding a single TMD and a short C-terminal tail (Abell and Mullen, 2011), can be targeted to the thylakoid membrane via the cpGET pathway. This pathway is assisted by targeting factor Get3B that binds the TMD of the TA protein in its hydrophobic groove (Anderson et al., 2021). The analog of the cpGET pathway in the cytoplasm, the GET pathway, is distributed throughout all eukaryotic cells and targets numerous TA proteins to the membranes exposed to the cytosol (Borgese et al., 2019). But thus far, the only known substrate of the cpGET pathway is CHLOROPLAST SECRETORY TRANSLOCASE E1 (cpSECE1), which appears to be sorted based on the characteristics of its TMD and C-terminal tail (Anderson et al., 2021). Further investigation is required to understand whether more substrates are translocated by the cpGET pathway and also the detailed regulatory mechanisms for protein targeting.

Some thylakoid membrane proteins are presumably inserted into the thylakoid membrane via the “spontaneous insertion” pathway, which is supposed to require no energy input or the assistance of other proteins for substrate insertion (Jarvis and Robinson, 2004; Schunemann, 2007). The proteins, such as THYLAKOID ASSEMBLY 4 (Tha4), HIGH CHLOROPHYLL FLUORESCENCE 106 (Hcf106), CF0 II, PsbX, PsbY, PsaG, and PsaK, are targeted by this pathway (Schleiff and Klösgen, 2001; Lee et al., 2017). However, spontaneous membrane insertion of these proteins could be only apparent: chaperones involved in the process or assembly into complexes with other subunits may actually occur but remain still unidentified. Examples include the Plsp1, a plastidic type I signal peptidase, which can insert into membrane spontaneously *in vitro* but is assisted by a large complex containing chaperonin 60 (Cpn60) in the stroma and cpSECY1 during insertion into thylakoid membranes (Endow et al., 2015; Klasek et al., 2020).

The thylakoid lumen proteins are translocated by the chloroplast secretory (cpSEC) pathway or the chloroplast twin-arginine translocase (cpTAT) pathway. These proteins carry an N-terminal bipartite targeting sequence, i.e., a standard transit peptide followed by a lumen targeting peptide (LTP) (Lee et al., 2017; Xu et al., 2021a). The cpSEC pathway involves the SECA1 ATPase and the thylakoid membrane-localized cpSECY1/E1 translocon, and tends to handle unfolded proteins (Albiniak et al., 2012; Fernandez, 2018). The cpTAT pathway comprises three membrane components, Hcf106, Tha4, and cpTatC, which work together as the translocon (Celedon and Cline, 2012; Ma et al., 2018). Compared to the cpSEC pathway, substrates of the cpTAT pathway are folded proteins or proteins assembled into complexes with co-factors, and their transmembrane translocation is promoted by the thylakoidal proton gradient (Jarvis and Lopez-Juez, 2013; New et al., 2018).

Besides the conformational differences, substrates of the cpSEC and cpTAT pathways have similar but different LTPs. Specifically, while LTPs of both pathways contain three distinct regions: an N-terminal charged region, a hydrophobic core, and a polar C-terminal domain, the cpTAT LTPs possess a twin arginine (RR) motif in the N-terminal region, which functions as the “cpSEC avoidance” motif (Fernandez, 2018; New et al., 2018). While the LTPs and the protein conformation required by the two lumen targeting pathways are quite clear, the underlying molecular mechanisms of targeting signal recognition and protein translocation through the aqueous stroma were almost completely unknown until recently, when Ouyang et al. (2020) revealed a novel mechanism for the cpTAT substrate sorting facilitated by STTs driven LLPS.

## Significance and Composition of Liquid Condensates

Liquid-liquid phase separation (LLPS) is a prevalent mechanism that drives intracellular membrane-less compartments formation across kingdoms of life and accelerates biochemical reactions spatiotemporally via concentrating macromolecules locally. In humans, animals, plants, and many other organisms, LLPS participates in various biological processes, such as ribosome biogenesis, gene expression, RNA processing, heterochromatin formation, etc. (Zhang et al., 2020; Emenecker et al., 2021; Kim et al., 2021; Xu et al., 2021b). Disturbing the process of LLPS may lead to liquid condensates vanishing or transforming into other material states, which are associated with many human diseases, including infectious diseases, neurodegeneration, and cancer (Alberti et al., 2019). Several liquid droplets appear to be plant specific, such as photobodies, which are sophisticated subnuclear condensates that robustly induce light signal transduction (Pardi and Nusinow, 2021), AUXIN RESPONSE FACTOR (ARF) condensates, which are cytoplasmic condensates regulating auxin transcriptional responses in Arabidopsis tissues that are no longer actively growing (Powers et al., 2019), and the pyrenoid, which is the liquid droplet for CO_2_ concentration and fixation found in the chloroplast of most unicellular algae (Freeman Rosenzweig et al., 2017).

Most liquid condensates formed via LLPS are composed of a heterogeneous mixture of macromolecules, and phase separation is driven by weak intermolecular forces between specific macromolecular components, based on multivalent protein-protein or protein-RNA interactions (Li et al., 2012; Emenecker et al., 2021). Emerging evidence indicates that proteins undergoing LLPS usually contain intrinsically disordered regions (IDRs) and low-complexity regions (LCRs) (Zhang et al., 2020). Intrinsically disordered regions fail to fold into a fixed three-dimensional structure but instead exhibit flexible and versatile conformations (Zhang et al., 2020). The net electric charge and hydrophilicity/hydrophobicity of IDRs are variable, due to the various biased amino acid compositions (Oldfield and Dunker, 2014). Some IDRs contain the LCR, a domain enriched in a specific subset of amino acid residues, such as poly-glycine, poly-serine, and poly-glutamine (Kato et al., 2012; Wright and Dyson, 2015). The flexible conformations of IDRs and the multitude of identical or highly similar residues in LCRs fulfill the requirement for weak multivalent interactions to drive LLPS (Posey et al., 2018).

## STTs Condensates and the cpTAT Pathway

In Arabidopsis, both STT1 and its homolog, STT2, are IDR-containing proteins. They interact specifically with cpTAT substrates OXYGEN EVOLVING COMPLEX SUBUNIT 23 kD (OE23), OE17, and the photosystem I subunit PSI-N (PsaN), but not with the cpSEC substrate OE33. Although STT1 and STT2 can physically interact with OE23 individually, together they promote the targeting and binding of iOE23 (the intermediate form of OE23 containing the LTP but without the transit peptide) to thylakoid membranes. This result was further confirmed by analyzing knock-down lines of STT1 and STT2. In this experiment, cpTAT specific substrates were dramatically reduced while other proteins were only moderately affected (Ouyang et al., 2020).

**FIGURE 1:**
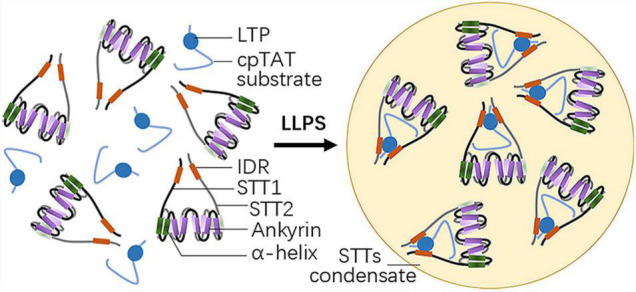
Structure of STT1/2 and LLPS driven by STT1/2. Both STT1 and STT2 have an N-terminal intrinsically disordered region (IDR) and five C-terminal ankyrin repeat domains following two α-helixes. STT1 and STT2 form an ellipsoidal-like core structure via the antiparallel interaction of their five ankyrin repeat domains and two adjacent N-terminal helixes. STT1 and STT2 complex binds the lumen targeting peptide (LTP) of the cpTat substrate by their IDRs and undergo liquid-liquid phase separation (LLPS).

STT1 and STT2 are two plant-specific proteins with an N-terminal IDR and five C-terminal ankyrin repeat domains (Figure 1). While individual STT1 or STT2 tends to aggregate, bimolecular fluorescence complementation (BiFC) experiments indicated STT1 directly interacts with STT2, facilitated by their ankyrin repeat domains to form a STTs complex. This is consistent with the results of crystal structure analysis, which showed that truncated STT1 and STT2, lacking the transit peptide and the IDR, form an ellipsoidal-like core structure through the antiparallel interaction of their two N-terminal helixes and five ankyrin repeat domains. Meanwhile, the conserved positively charged residues within the ankyrin repeat domains are crucial for STTs oligomer formation (Ouyang et al., 2020).

Pull-down assays, isothermal titration calorimetry (ITC) analyses, together with residue-substitution mutation assays showed that the STTs complex binds the cpTAT substrate OE23 or its LTP at an approximately 1:1 protein: protein or protein: peptide ratio, and that both the RR residues in the N-terminal charged region and the hydrophobic core of OE23 LTP, as well as the negatively charged residues of the WEEPD motif in the STT1 IDR and the hydrophobic residues of the LVP-W motif in the STT2 IDR, are required for STTs-OE23 heterotrimers formation and subsequent cpTAT substrate targeting (Ouyang et al., 2020).

Considering that STT1 and STT2 contain IDRs, the STTs complexes may undergo LLPS. As speculated, microscopy imaging assays and photobleaching showed that STTs complexes lead to spherical liquid condensates through LLPS at high concentration *in vitro*, when mixed with OE23 LTPs at the ratio of 1:1 (Figure 1). STTs complex formation is a prerequisite for LLPS, as shown by the observations that liquid condensates after mixing with LTPs do not form when individual STT1 or STT2 are used or when the STTs complex formation is disrupted by mutating the electropositive residues within the ankyrin repeat domains. The STTs-LTP binding reaction is also required for LLPS, as substitution of the residues of the LTP binding motif within the STTs, such as the RR residues in the N-terminal charged region and residues in the hydrophobic core, severely compromised liquid condensates formation. Thus, multivalent interactions and hydrophobic interactions between STT1, STT2, and LTP drive LLPS and form STTs condensates (Ouyang et al., 2020).

BiFC and pull-down assays further revealed that STT1 and STT2 interact with the stromal domain of cpTAT translocon component Hcf106. Binding to Hcf106 hinders STTs oligomerization and LLPS, thereby releasing and docking cpTAT substrates to the cpTatC-Hcf106 receptor complex and facilitating substrate translocation across the thylakoid membrane (Ouyang et al., 2020).

In general, the STT1 and STT2 complex undergoes cpTAT substrate-induced LLPS and form the STTs condensates to facilitate the substrate targeting and translocation into the thylakoid lumen. Along with the STTs, many other targeting factors were predicted to contain IDRs. These factors include but are not limited to the following (Ouyang et al., 2020): (1) SECA1 in Arabidopsis and rice, and SECA2 in Arabidopsis but not rice. These proteins are assistants of substrate translocation to the thylakoid lumen and the inner chloroplast envelope, respectively; (2) cpSRP43 and cpSRP54 in Arabidopsis and rice. These proteins are the components of the cpSRP pathway; (3) SRP54 in *Escherichia coli*, yeast, Arabidopsis and mice. This protein is a cytosolic translocation factor for secreted proteins; (4) PEX5 and PEX13 in Arabidopsis and mice. These proteins are components of the peroxisomal protein translocation machinery; (5) AKR2A in Arabidopsis and mice. This protein is a cytosolic targeting factor. As IDR-containing proteins have been widely implicated in mediating phase separation (Li et al., 2012; Molliex et al., 2015), these factors mentioned above probably undergo LLPS and lead to liquid condensate formation, implicating that LLPS may be a universal and conserved mechanism for substrate sorting not only in the chloroplast of the plant but also in the cytoplasm across kingdoms (Lee and Hwang, 2020; Ouyang et al., 2020).

## Perspectives

Within cells, LLPS is tightly regulated by various mechanisms for proper functions. Physical conditions that can change affinities of biomolecular multivalent interactions, such as pH, temperature, redox state, ionic strength, and osmotic pressure, are known to have an effect on LLPS of biomolecular systems (Banani et al., 2017). Within the chloroplast, the redox state, which interplays with the photosynthetic light reactions, fluctuates rapidly with light intensity changes, biotic and abiotic stresses suffered, diurnal variation, and other environmental stimuli (Foyer, 2018; Kuzniak and Kopczewski, 2020; Sachdev et al., 2021). And the stromal pH oscillates during light-dark transition. In the dark both the cytoplasmic and stromal pH is close to 7, but upon illumination the stroma becomes alkaline and the pH increases to about 8.0, as a consequence of H^+^-pumping into the thylakoid lumen (Heldt et al., 1973; Hohner et al., 2016). In addition, dynamics of many ions in the stroma have been observed in response to stress stimuli and also at the transition between light and darkness, especially Mg^2+^ and Ca^2+^, which play a critical role in regulating enzyme activity and other numerous physiological and biochemical processes (Szabo and Spetea, 2017; Frank et al., 2019; Li et al., 2020; Marti Ruiz et al., 2020). In summary, physical conditions within the chloroplast change frequently through variations of environmental and metabolic conditions. In addition, various post-translational modifications alter the interactions among macromolecules and can modulate LLPS (Bah and Forman-Kay, 2016; Zhang et al., 2020). While post-translational modifications in the chloroplasts have been poorly investigated compared to whole plant cells, animal cells, and yeast, numerous types of post-translational modification have been identified and are believed to occur in the chloroplast, such as phosphorylation, oxidation-reduction, acetylation, methylation, and glutathionylation (Lehtimaki et al., 2015; Grabsztunowicz et al., 2017). However, whether or not STTs driven LLPS is regulated by any physical condition changes or post-translational modifications within the chloroplast is still unclear. Further studies on this topic will expand our knowledge of the regulation of intra-chloroplast protein sorting.

The TAT system has been found in prokaryotes, chloroplasts, and some mitochondria, allowing folded proteins to be transported across membranes (Berks, 2015). The TAT pathway in both chloroplasts and bacteria comprise similar membrane located translocon components, cpTatC (TatC), Hcf106 (TatB), and Tha4 (TatA) (New et al., 2018), but each has different chaperones. In bacteria, some substrates of TAT pathway have specific cytosolic chaperones, whereas some are assisted by the general chaperones, such as DnaK and DnaJ in *E. coli* (Robinson et al., 2011). In Arabidopsis, cpTAT passenger proteins are assisted by the general chaperones, STT1 and STT2. However, orthologs of STTs could be identified only in the angiosperm and gymnosperm lineages, and none was detected in animals, microorganisms, or even other photosynthetic species including cyanobacteria, eukaryotic unicellular algae and mosses (Garcion et al., 2006), indicating STTs-driven LLPS as an emerging mechanism for cpTAT substrate sorting during plant evolution. Phylogenetic analyses should be taken to find the origin of STTs and chaperones for cpTAT pathway in lower plants need to be identified, which are important challenges for the future to decipher the sorting process and the mechanism for regulating the cpTAT pathway.

## Author Contributions

CZ and DL led the writing effort with substantial contributions from XX and LZ. All the authors read and approved the submitted version.

## Conflict of Interest

The authors declare that the research was conducted in the absence of any commercial or financial relationships that could be construed as a potential conflict of interest.

## Publisher’s Note

All claims expressed in this article are solely those of the authors and do not necessarily represent those of their affiliated organizations, or those of the publisher, the editors and the reviewers. Any product that may be evaluated in this article, or claim that may be made by its manufacturer, is not guaranteed or endorsed by the publisher.

## References

[B1] Abell B. M. Mullen R. T. (2011). Tail-anchored membrane proteins: exploring the complex diversity of tail-anchored-protein targeting in plant cells. *Plant Cell Rep.* 30 137–151. 10.1007/s00299-010-0925-6 20878326

[B2] Alberti S. (2017). Phase separation in biology. *Curr. Biol.* 27 R1097–R1102. 10.1016/j.cub.2017.08.069 29065286

[B3] Alberti S. Gladfelter A. Mittag T. (2019). Considerations and challenges in studying liquid-liquid phase Sseparation and biomolecular condensates. *Cell* 176 419–434. 10.1016/j.cell.2018.12.035 30682370 PMC6445271

[B4] Albiniak A. M. Baglieri J. Robinson C. (2012). Targeting of lumenal proteins across the thylakoid membrane. *J. Exp. Bot.* 63 1689–1698. 10.1093/jxb/err444 22275386

[B5] Anderson S. A. Satyanarayan M. B. Wessendorf R. L. Lu Y. Fernandez D. E. (2021). A homolog of GuidedEntry of Tail-anchored proteins3 functions in membrane-specific protein targeting in chloroplasts of Arabidopsis. *Plant Cell.* 33 2812–2833. 10.1093/plcell/koab145 34021351 PMC8408437

[B6] Bah A. Forman-Kay J. D. (2016). Modulation of intrinsically disordered protein function by post-translational modifications. *J. Biol. Chem.* 291 6696–6705. 10.1074/jbc.R115.695056 26851279 PMC4807257

[B7] Banani S. F. Lee H. O. Hyman A. A. Rosen M. K. (2017). Biomolecular condensates: organizers of cellular biochemistry. *Nat. Rev. Mol. Cell. Biol.* 18 285–298. 10.1038/nrm.2017.7 28225081 PMC7434221

[B8] Berks B. C. (2015). The twin-arginine protein translocation pathway. *Annu. Rev. Biochem.* 84 843–864. 10.1146/annurev-biochem-060614-034251 25494301

[B9] Borgese N. Coy-Vergara J. Colombo S. F. Schwappach B. (2019). The ways of Tails: the GET pathway and more. *Protein J.* 38 289–305. 10.1007/s10930-019-09845-4 31203484

[B10] Bouchnak I. Brugiere S. Moyet L. Le Gall S. Salvi D. Kuntz M. (2019). Unraveling hidden components of the chloroplast envelope proteome: opportunities and limits of better MS sensitivity. *Mol. Cell Proteomics* 18 1285–1306. 10.1074/mcp.RA118.000988 30962257 PMC6601204

[B11] Brangwynne C. P. Eckmann C. R. Courson D. S. Rybarska A. Hoege C. Gharakhani J. (2009). Germline P granules are liquid droplets that localize by controlled dissolution/condensation. *Science* 324 1729–1732. 10.1126/science.1172046 19460965

[B12] Bruce B. D. (2000). Chloroplast transit peptides: structure, function and evolution. *Trends Cell. Biol.* 10 440–447. 10.1016/s0962-8924(00)01833-x 10998602

[B13] Celedon J. M. Cline K. (2012). Stoichiometry for binding and transport by the twin arginine translocation system. *J. Cell. Biol.* 197 523–534. 10.1083/jcb.201201096 22564412 PMC3352945

[B14] Emenecker R. J. Holehouse A. S. Strader L. C. (2021). Biological phase separation and biomolecular condensates in plants. *Annu. Rev. Plant Biol.* 72 17–46. 10.1146/annurev-arplant-081720-015238 33684296 PMC8221409

[B15] Endow J. K. Singhal R. Fernandez D. E. Inoue K. (2015). Chaperone-assisted post-translational transport of plastidic type I signal peptidase 1. *J. Biol. Chem.* 290 28778–28791. 10.1074/jbc.M115.684829 26446787 PMC4661394

[B16] Falk S. Sinning I. (2010). cpSRP43 is a novel chaperone specific for light-harvesting chlorophyll a,b-binding proteins. *J. Biol. Chem.* 285 21655–21661. 10.1074/jbc.C110.132746 20498370 PMC2898393

[B17] Fernandez D. E. (2018). Two paths diverged in the stroma: targeting to dual SEC translocase systems in chloroplasts. *Photosynth Res.* 138 277–287. 10.1007/s11120-018-0541-9 29951837

[B18] Foyer C. H. (2018). Reactive oxygen species, oxidative signaling and the regulation of photosynthesis. *Environ. Exp. Bot.* 154 134–142. 10.1016/j.envexpbot.2018.05.003 30283160 PMC6105748

[B19] Frank J. Happeck R. Meier B. Hoang M. T. T. Stribny J. Hause G. (2019). Chloroplast-localized BICAT proteins shape stromal calcium signals and are required for efficient photosynthesis. *New Phytol.* 221 866–880. 10.1111/nph.15407 30169890

[B20] Freeman Rosenzweig E. S. Xu B. Kuhn Cuellar L. Martinez-Sanchez A. Schaffer M. Strauss M. (2017). The eukaryotic CO2-concentrating organelle is liquid-like and exhibits dynamic reorganization. *Cell* 171, 148–162.e19. 10.1016/j.cell.2017.08.008 28938114 PMC5671343

[B21] Garcion C. Guilleminot J. Kroj T. Parcy F. Giraudat J. Devic M. (2006). AKRP and EMB506 are two ankyrin repeat proteins essential for plastid differentiation and plant development in Arabidopsis. *Plant J.* 48 895–906. 10.1111/j.1365-313X.2006.02922.x 17092312

[B22] Grabsztunowicz M. Koskela M. M. Mulo P. (2017). Post-translational modifications in regulation of chloroplast function: recent advances. *Front. Plant Sci.* 8:240. 10.3389/fpls.2017.00240 28280500 PMC5322211

[B23] Heldt H. W. Werdan K. Milovancev M. Geller G. (1973). Alkalization of the chloroplast stroma caused by light-dependent proton flux into the thylakoid space. *Biochim. Biophys. Acta* 314 224–241. 10.1016/0005-2728(73)90137-0 4747067

[B24] Hohner R. Aboukila A. Kunz H. H. Venema K. (2016). Proton gradients and proton-dependent transport processes in the chloroplast. *Front. Plant Sci.* 7:218. 10.3389/fpls.2016.00218 26973667 PMC4770017

[B25] Jarvis P. Lopez-Juez E. (2013). Biogenesis and homeostasis of chloroplasts and other plastids. *Nat. Rev. Mol. Cell. Biol.* 14 787–802. 10.1038/nrm3702 24263360

[B26] Jarvis P. Robinson C. (2004). Mechanisms of protein import and routing in chloroplasts. *Curr. Biol.* 14 R1064–R1077. 10.1016/j.cub.2004.11.049 15620643

[B27] Kato M. Han T. W. Xie S. Shi K. Du X. Wu L. C. (2012). Cell-free formation of RNA granules: low complexity sequence domains form dynamic fibers within hydrogels. *Cell* 149 753–767. 10.1016/j.cell.2012.04.017 22579281 PMC6347373

[B28] Kim J. Lee H. Lee H. G. Seo P. J. (2021). Get closer and make hotspots: liquid-liquid phase separation in plants. *EMBO Rep.* 22:e51656. 10.15252/embr.202051656 33913240 PMC8097390

[B29] Kirchhoff H. (2019). Chloroplast ultrastructure in plants. *New Phytol.* 223 565–574. 10.1111/nph.15730 30721547

[B30] Klasek L. Inoue K. Theg S. M. (2020). Chloroplast chaperonin-mediated targeting of a thylakoid membrane protein. *Plant Cell* 32 3884–3901. 10.1105/tpc.20.00309 33093145 PMC7721336

[B31] Kuzniak E. Kopczewski T. (2020). The chloroplast reactive oxygen species-redox system in plant immunity and disease. *Front. Plant. Sci.* 11:572686. 10.3389/fpls.2020.572686 33281842 PMC7688986

[B32] Lee D. W. Hwang I. (2020). Liquid-liquid phase transition as a new means of protein targeting in chloroplasts. *Mol. Plant* 13 679–681. 10.1016/j.molp.2020.04.001 32298786

[B33] Lee D. W. Hwang I. (2021). Understanding the evolution of endosymbiotic organelles based on the targeting sequences of organellar proteins. *New Phytol.* 230 924–930. 10.1111/nph.17167 33404103

[B34] Lee D. W. Lee J. Hwang I. (2017). Sorting of nuclear-encoded chloroplast membrane proteins. *Curr. Opin. Plant Biol.* 40 1–7. 10.1016/j.pbi.2017.06.011 28668581

[B35] Lehtimaki N. Koskela M. M. Mulo P. (2015). Posttranslational modifications of chloroplast proteins: an emerging field. *Plant Physiol.* 168 768–775. 10.1104/pp.15.00117 25911530 PMC4741338

[B36] Li J. Yokosho K. Liu S. Cao H. R. Yamaji N. Zhu X. G. (2020). Diel magnesium fluctuations in chloroplasts contribute to photosynthesis in rice. *Nat Plants* 6 848–859. 10.1038/s41477-020-0686-3 32541951

[B37] Li P. Banjade S. Cheng H. C. Kim S. Chen B. Guo L. (2012). Phase transitions in the assembly of multivalent signalling proteins. *Nature* 483 336–340. 10.1038/nature10879 22398450 PMC3343696

[B38] Li Y. Martin J. R. Aldama G. A. Fernandez D. E. Cline K. (2017). Identification of putative substrates of SEC2, a chloroplast inner envelope translocase. *Plant Physiol.* 173 2121–2137. 10.1104/pp.17.00012 28213560 PMC5373066

[B39] Li Y. Singhal R. Taylor I. W. McMinn P. H. Chua X. Y. Cline K. (2015). The Sec2 translocase of the chloroplast inner envelope contains a unique and dedicated SECE2 component. *Plant J.* 84 647–658. 10.1111/tpj.13028 26406904

[B40] Ma Q. Fite K. New C. P. Dabney-Smith C. (2018). Thylakoid-integrated recombinant Hcf106 participates in the chloroplast twin arginine transport system. *Plant Direct* 2:e00090. 10.1002/pld3.90 31245690 PMC6508782

[B41] Marti Ruiz M. C. Jung H. J. Webb A. A. R. (2020). Circadian gating of dark-induced increases in chloroplast- and cytosolic-free calcium in Arabidopsis. *New Phytol.* 225 1993–2005. 10.1111/nph.16280 31644821 PMC7028143

[B42] Molliex A. Temirov J. Lee J. Coughlin M. Kanagaraj A. P. Kim H. J. (2015). Phase separation by low complexity domains promotes stress granule assembly and drives pathological fibrillization. *Cell* 163 123–133. 10.1016/j.cell.2015.09.015 26406374 PMC5149108

[B43] New C. P. Ma Q. Dabney-Smith C. (2018). Routing of thylakoid lumen proteins by the chloroplast twin arginine transport pathway. *Photosynth Res.* 138 289–301. 10.1007/s11120-018-0567-z 30101370

[B44] Oldfield C. J. Dunker A. K. (2014). Intrinsically disordered proteins and intrinsically disordered protein regions. *Annu. Rev. Biochem.* 83 553–584. 10.1146/annurev-biochem-072711-164947 24606139

[B45] Ouyang M. Li X. Ma J. Chi W. Xiao J. Zou M. (2011). LTD is a protein required for sorting light-harvesting chlorophyll-binding proteins to the chloroplast SRP pathway. *Nat. Commun.* 2:277. 10.1038/ncomms1278 21505433

[B46] Ouyang M. Li X. Zhang J. Feng P. Pu H. Kong L. (2020). Liquid-liquid phase transition drives intra-chloroplast cargo sorting. *Cell* 180, 1144–1159.e20. 10.1016/j.cell.2020.02.045 32169217

[B47] Paila Y. D. Richardson L. G. L. Schnell D. J. (2015). New insights into the mechanism of chloroplast protein import and its integration with protein quality control, organelle biogenesis and development. *J. Mol. Biol.* 427 1038–1060. 10.1016/j.jmb.2014.08.016 25174336 PMC4339491

[B48] Pardi S. A. Nusinow D. A. (2021). Out of the dark and into the light: a new view of phytochrome photobodies. *Front. Plant Sci.* 12:732947. 10.3389/fpls.2021.732947 34531891 PMC8438518

[B49] Posey A. E. Holehouse A. S. Pappu R. V. (2018). Phase separation of intrinsically disordered proteins. *Methods Enzymol.* 611 1–30. 10.1016/bs.mie.2018.09.035 30471685

[B50] Powers S. K. Holehouse A. S. Korasick D. A. Schreiber K. H. Clark N. M. Jing H. (2019). Nucleo-cytoplasmic partitioning of ARF proteins controls auxin responses in Arabidopsis thaliana. *Mol. Cell* 76, 177–190.e5. 10.1016/j.molcel.2019.06.044 31421981 PMC6778021

[B51] Richardson L. G. Paila Y. D. Siman S. R. Chen Y. Smith M. D. Schnell D. J. (2014). Targeting and assembly of components of the TOC protein import complex at the chloroplast outer envelope membrane. *Front. Plant Sci.* 5:269. 10.3389/fpls.2014.00269 24966864 PMC4052903

[B52] Robinson C. Matos C. F. Beck D. Ren C. Lawrence J. Vasisht N. (2011). Transport and proofreading of proteins by the twin-arginine translocation (Tat) system in bacteria. *Biochim. Biophys. Acta* 1808 876–884. 10.1016/j.bbamem.2010.11.023 21126506

[B53] Sachdev S. Ansari S. A. Ansari M. I. Fujita M. Hasanuzzaman M. (2021). Abiotic stress and reactive oxygen species: generation, signaling, and defense mechanisms. *Antioxidants* 10:277. 10.3390/antiox10020277 33670123 PMC7916865

[B54] Schleiff E. Klösgen R. B. (2001). Without a little help from ‘my’ friends: direct insertion of proteins into chloroplast membranes? *Biochim. Biophys. Acta* 1541 22–33. 10.1016/s0167-4889(01)00152-5 11750660

[B55] Schunemann D. (2007). Mechanisms of protein import into thylakoids of chloroplasts. *Biol. Chem.* 388 907–915. 10.1515/BC.2007.111 17696774

[B56] Shin Y. Brangwynne C. P. (2017). Liquid phase condensation in cell physiology and disease. *Science* 357:eaaf4382. 10.1126/science.aaf4382 28935776

[B57] Stengel K. F. Holdermann I. Cain P. Robinson C. Wild K. Sinning I. (2008). Structural basis for specific substrate recognition by the chloroplast signal recognition particle protein cpSRP43. *Science* 321 253–256. 10.1126/science.1158640 18621669

[B58] Szabo I. Spetea C. (2017). Impact of the ion transportome of chloroplasts on the optimization of photosynthesis. *J. Exp. Bot.* 68 3115–3128. 10.1093/jxb/erx063 28338935

[B59] Viana A. A. Li M. Schnell D. J. (2010). Determinants for stop-transfer and post-import pathways for protein targeting to the chloroplast inner envelope membrane. *J. Biol. Chem.* 285 12948–12960. 10.1074/jbc.M110.109744 20194502 PMC2857071

[B60] Wright P. E. Dyson H. J. (2015). Intrinsically disordered proteins in cellular signalling and regulation. *Nat. Rev. Mol. Cell. Biol.* 16 18–29. 10.1038/nrm3920 25531225 PMC4405151

[B61] Xu X. Ouyang M. Lu D. Zheng C. Zhang L. (2021a). Protein sorting within chloroplasts. *Trends Cell. Biol.* 31 9–16. 10.1016/j.tcb.2020.09.011 33121860

[B62] Xu X. Zheng C. Lu D. Song C. P. Zhang L. (2021b). Phase separation in plants: new insights into cellular compartmentalization. *J. Integr. Plant Biol.* 63 1835–1855. 10.1111/jipb.13152 34314106

[B63] Zhang H. Ji X. Li P. Liu C. Lou J. Wang Z. (2020). Liquid-liquid phase separation in biology: mechanisms, physiological functions and human diseases. *Sci. China Life Sci.* 63 953–985. 10.1007/s11427-020-1702-x 32548680

[B64] Ziehe D. Dunschede B. Schunemann D. (2018). Molecular mechanism of SRP-dependent light-harvesting protein transport to the thylakoid membrane in plants. *Photosynth Res.* 138 303–313. 10.1007/s11120-018-0544-6 29956039 PMC6244792

